# Regenerated Cellulose Products for Agricultural and Their Potential: A Review

**DOI:** 10.3390/polym13203586

**Published:** 2021-10-18

**Authors:** Nur Amira Zainul Armir, Amalia Zulkifli, Shamini Gunaseelan, Swarna Devi Palanivelu, Kushairi Mohd Salleh, Muhamad Hafiz Che Othman, Sarani Zakaria

**Affiliations:** 1 Bioresources and Biorefinery Laboratory, Department of Applied Physics, Faculty of Science and Technology, University Kebangsaan Malaysia, Bangi 43600, Selangor, Malaysia; amirazainularmir@gmail.com (N.A.Z.A.); amaliaxzulkifli@gmail.com (A.Z.); shamini_88@hotmail.com (S.G.); P109377@siswa.ukm.edu.my (S.D.P.); 2 Department of Biological Sciences and Biotechnology, Faculty of Science and Technology, Universiti Kebangsaan Malaysia, Bangi 43600, Selangor, Malaysia; hafiz87@ukm.edu.my

**Keywords:** fertilizer, mulching film, smart farming, soilless farming, water holding capacity

## Abstract

Cellulose is one of the most abundant natural polymers with excellent biocompatibility, non-toxicity, flexibility, and renewable source. Regenerated cellulose (RC) products result from the dissolution-regeneration process risen from solvent and anti-solvent reagents, respectively. The regeneration process changes the cellulose chain conformation from cellulose I to cellulose II, leads the structure to have more amorphous regions with improved crystallinity, and inclines towards extensive modification on the RC products such as hydrogel, aerogel, cryogel, xerogel, fibers, membrane, and thin film. Recently, RC products are accentuated to be used in the agriculture field to develop future sustainable agriculture as alternatives to conventional agriculture systems. However, different solvent types and production techniques have great influences on the end properties of RC products. Besides, the fabrication of RC products from solely RC lacks excellent mechanical characteristics. Thus, the flexibility of RC has allowed it to be homogenously blended with other materials to enhance the final products’ properties. This review will summarize the properties and preparation of potential RC-based products that reflect its application to replace soil the plantation medium, govern the release of the fertilizer, provide protection on crops and act as biosensors.

## 1. Introduction

The conventional agricultural or known as the industrial agriculture system, is regarded as non-sustainable subjected to how it is being governed and practiced. It is known as an agricultural system that uses synthetic fertilizers, herbicides, and pesticides and implements crop rotation [1]. Fertilizers are beneficial to improve the yield quantity; however, the long-term use of fertilizer has caused environmental non-point source (NPS) pollution [2]. Alongside, the conventional agricultural system is inexorably inefficient, subject to the chemicals’ usage implications, soil degradation, large-scale plantations, crops’ loss due to diseases and pests, less profitability, jeopardizing humans and crops’ health [1,3,4]. The conventional agricultural practice also causes the degradation in water quality simultaneously uses up a huge amount of water. The water from any possible and suitable sources will be withdrawn and supplied to the irrigated crops, which concurrently hoisting the salt from soils and the contaminants elicited from the usage of fungicides, pesticides, and fertilizers [5]. The water from the irrigated croplands will flow back to the streams and, in conjunction with land loss due to urbanization and residential expansion, surge the water salinization, waterlogging, river waters’ reduction, and loss of soil moisture [6,7]. Ensuring a sustainable world from the agriculture perspective has motivated the usage of sustainable and biodegradable materials as alternatives.

The abundance of natural polysaccharides such as cellulose, pectin, alginate, starch, chitosan, xanthan gum is widely used as biopolymer materials and has benefited human beings for ages. Polysaccharides are also referred to as biopolymers that consist of approximately >20 to 60,000 monosaccharides units and are known as part of the plant cell wall, algae origin, animal origin, and bacterial production. The complex structure of polysaccharides has turned them into valuable elements on earth because they provide interesting interaction among themselves and other components such as protein [8]. The excellent characteristics of polysaccharides such as sustainable, cheap, and edible have made them popular biomaterials that are fabricated into coatings [9], films [10], hydrogels [11], and fibers [12]. Recently, the idea of using polysaccharide-inspired products for agriculture purposes has emerged as a modern way to overcome the issues related to conventional agriculture. The flexibility and precious properties such as possessing hydroxyl groups and hydrogen bonding between the hydroxyl groups made them water-soluble, rigid, high tensile strength, and viscous [8]. For instance, chitosan-films were employed in the plantation soil to control the soil microbiota, remediation, fertilizer-release system, and water absorbency [13]. Grafted sodium-alginate superabsorbent hydrogel with poly(acrylic) acid that exhibited the ability to release sodium humate was used to facilitate plant growth by accelerating the root development [14]. Plant-derived biomass such as lignocellulosic biomass has the potential to become a great resource as it offers a sustainable manner when processed into products [15,16]. Cellulose, a part of lignocellulosic biomass among lignin, hemicellulose, and cellulose derivatives, has shown great potential due to its advantageous properties that will be discussed in the following paragraphs.

**Figure 1:**
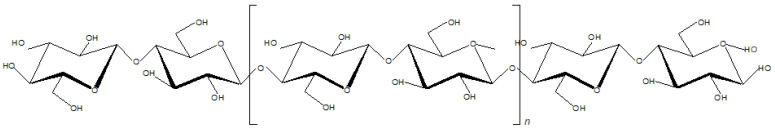
Structure of cellulose.

Cellulose was first mentioned by a French chemist named Anselme Payen in 1838 when he obtained a ‘residue’ resulted from the acids and ammonia treatment on different parts of plant tissues as well as performing water extraction procedures [17]. It is a hydrophilic linear homopolymer that has a molecular formula of (C_6_H_10_O_5_)*_n_* and is made up of anhydroglucose (AGU) monomers or also known as *D*-glucose or glucans units [18]. AGU monomers are covalently bonded and formed the *ß*-1,4-glycosidic bonds between C1 of one glucose ring and C4 of another glucose ring, stabilized by the bond formation between –OH and oxygen [19] (Figure 1). Cellulose remarkably unveils a range of versatile applications and is commercially marketed aside from its nature being an essential element for plant cell structure and development. It is notably employed in numerous applications such as medical and pharmaceutical, agricultural, cosmetics, sensor technology, water treatment, packaging, textiles, electrical devices, and food technology fields because it is low-cost, a renewable polymer, abundantly available, biodegradable, biocompatible, and harmless to the environment. Cellulose is regarded as one of the most important polymeric materials as it holds a unique structural architecture that brings specificity for the interaction with other molecules.

Cellulose I or known as native cellulose, exists as cellulose I_α_ and I_β_ allomorphs that found the majority in higher plants for the former while algae and bacteria for the latter [20]. The hydrogen bonding that exists via intra- and intermolecular interaction along the repeating units of glycosidic-β-1,4-bonded-anyhydroglucose units is a major determinant of its behavior. Higher intermolecular and intramolecular hydrogen bonding dictate the properties of cellulose on account of a highly packed structure that eventually leads to higher crystallinity and thermal stability and stabilization and inhibits the thermal expansion, respectively [20,21]. The different plants will produce a different molecular weight of cellulose, whereby the molecular weight is manifested as one of the crucial factors that affect the cellulose solubility that eventually determines its solubility for application and development understanding [22]. The higher molecular weight of cellulose causes lower solubility of cellulose and vice versa based on higher molecular weight lowers the activation energy during cellulose dissolution. Another aspect that influences the solubility of cellulose is known as crystallinity that accounts for the crystalline area of cellulose; various measurements have been performed to determine the crystallinity index of cellulose [23]. Reduction in crystallinity of cellulose could increase the cellulose solubility; the structure of cellulose is not fully understood as its complex structure does not only revolve around crystallinity and amorphous aspects [23].

Native cellulose is challenging to be processed into desired products because of its insolubility in many solvents; thus, it needs to undergo powerful solvent treatment to break its supramolecular structure to dissolve it [24]. In recent years, the development of a deep understanding of cellulose regeneration drives the regenerated cellulose (RC) products to be used in the sustainable agriculture system, thus inspiring this review. This review aims to emphasize the applicability of RC products to be used in future agriculture systems and practice and to assist the readers and widen the attention for sustainable agriculture research. By definition, cellulose regeneration involves a structural transformation from cellulose I to cellulose II via two core techniques that are summarized as derivatizing and non-derivatizing processes [25]. The major fundamental difference between these two processes is the formation of modified cellulose preceding the regeneration process, where in this case, reflected by the derivatizing process while non-derivatizing solvents cause covalent modification is introduced by interfering with the inter- and intramolecular hydrogen molecular bonding. Correspond to this, while direct dissolution process employs physical intermolecular interactions (e.g., ionic liquids (ILs), NMMO, alkali-based solvents) then followed by precipitation with anti-solvent induction (e.g., coagulants, water) [25,26,27,28,29]. The regeneration is triggered to operate when the aqueous cellulose is energetically deprived for molecular dispersion and shifts the transient monomolecular sheets of hydrophobic interaction of glucopyranose rings of cellulose to the hydrogen-bonded that form cellulose II arrangement [30,31]. The regeneration is a counter-diffusion process between solvent and anti-solvents that reforms the inter- and intra-molecular hydrogen bonding in the cellulose structure [12].

The derivatizing process allows the formation of cellulose derivatives that give intermediate compounds such as ‘unstable’ ether-, ester-, intermediate acetal- upon dissolving in derivatizing solvents such as fluoroacetic acid, formate acid, dimethylsulfoxide (DMSO), and dimethylformamide (DMF) [32]. A non-derivatizing solvent such as alkali/urea aqueous system that is commonly used, cellulose is dissolved as the alkali hydrates, and urea complexes formation has contributed to this system [32]. A new hydrogen bond is formed when alkali hydrates tend to join the hydroxyl groups of cellulose. Then, the formation of inclusion complexes occurs due to the interaction between urea and alkali ions that enables the permeation of water molecules and alkali hydrates into the crystals of cellulose. It aids the process of dissolving subsequently. Next, for non-aqueous systems in this category, as an example, ILs are commonly used because they offer various advantages such as environmental-friendly solvent and possess excellent thermal and chemical stability [33]. The conformational change from cellulose I to cellulose II was observed upon dissolving cellulose in ILs that is triggered by anion and superbase cations of ILs bonded to the polar and non-polar domains of cellulose by Van der Waals forces, respectively [34]. The exceptional ability to dissolve cellulose by ILs is due to the lower degree of polymerization of cellulose imposed by easy aggregation of cellulose chains and, subsequently, regenerated in anti-solvent system [35]. Despite outstanding properties and abilities of ILs producing RC, a derivatizing aqueous system using alkali/urea solvent is preferred because some inflicted disadvantages of ILs such as expensive as well as separation and purification process to obtain RC [36]. The examples of cellulose solvents and their mechanism are summarized in Table 1.

**Figure 2:**
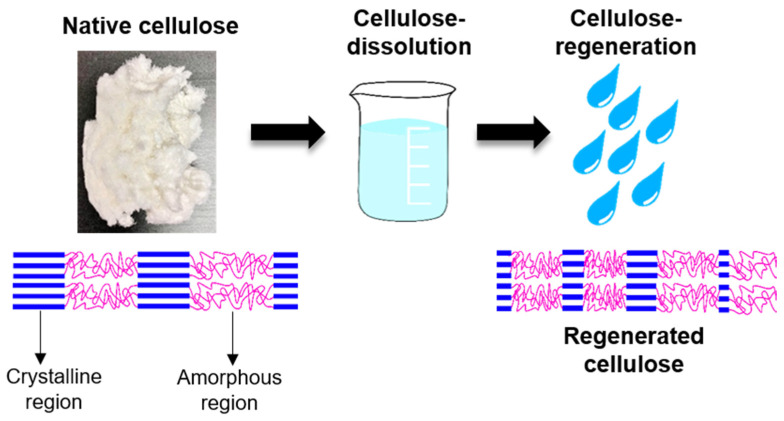
The dissolution-regeneration of native cellulose.

**Figure 3:**
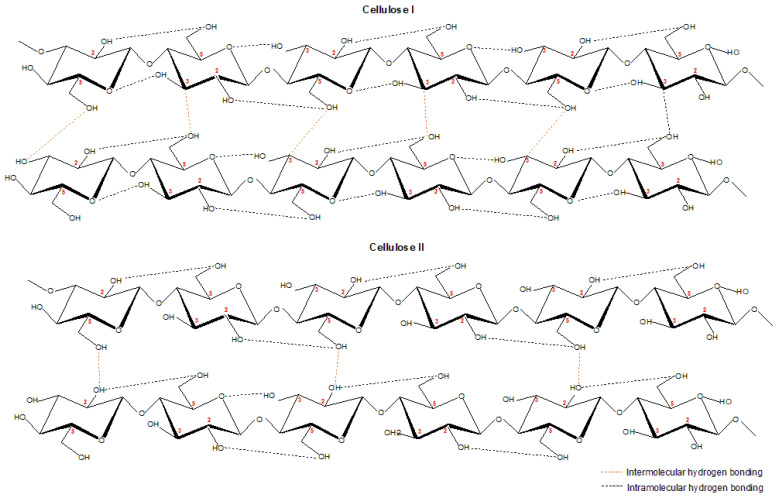
The structure of cellulose I and cellulose II.

The dissolution of cellulose I transforms the cellulose allomorph into cellulose II that is known as the most stable allormorph of cellulose, as illustrated in Figure 2. In terms of structure orientation, cellulose I and II are observed to be in parallel and antiparallel orientation, respectively. The difference between cellulose I and II in term of their inter- and intramolecular hydrogen bonding are shown if Figure 3. Cellulose I has inter- and intramolecular hydrogen bonding at O3H---O5′ and O6H---O3′, respectively, while O2H---O6′ (which also linked in cellulose I) and O6H---O2′are reported to be intra- and intermolecular hydrogen bonding for cellulose II [38]. Cellulose II has a low degree of polymerization, low elastic modulus, high polydispersity, and reduced crystallinity that causes its decomposition at a lower temperature as compared to cellulose I [26,39]. Although the crystallinity of regenerated cellulose is dictated by its dissolving solvent, studies have shown a reduction in crystallinity of regenerated cellulose. The transformation of cellulose I to regenerated cellulose can be observed via X-ray diffractogram, infrared profile, ^13^C nuclear magnetic radioactive (NMR) spectra, and thermal analyses. The aforementioned examples about the transformation characterization of cellulose I to cellulose II (or RC) are listed in Table 2. The regeneration of cellulose, which is a favored process, has been widely explored due to the crystallinity improvisations it brings on the properties of cellulose [40,41].

## 2. Regenerated Cellulose Products

### 2.1. Hydrogel, Aerogel, Cryogel, and Xerogel

Generally, hydrogel is a three-dimensional cross-linked polymeric network and able to absorb and retain a large volume of water due to extensive hydrophilic chains of the polymers employed such as –OH, -NH_2,_ -COOH, and –SO_3_H that is associated with the polymeric backbones [52,53,54]. Physical cross-linking is formed via various mechanisms include ionic interaction, hydrophobic interaction, hydrogen bonding, ultrasonic induction, and crystallization or classified as non-covalent interactions [55,56,57]. The chemical cross-linking occurs as the chain-growth polymerization, irradiation, covalent bonding, step-growth polymerization ‘click’, and chemistry that induce the covalent interaction. The cross-linking actions determine the properties of a hydrogel, such as a brittle and rigid hydrogel is formed via higher cross-linking density, and low cross-linking density manifests a good elasticity hydrogel. Some other products can be extended from hydrogel to broaden the applications by varying the drying method or liquid removal from the hydrogel afterward (Table 3).

**Figure 4:**
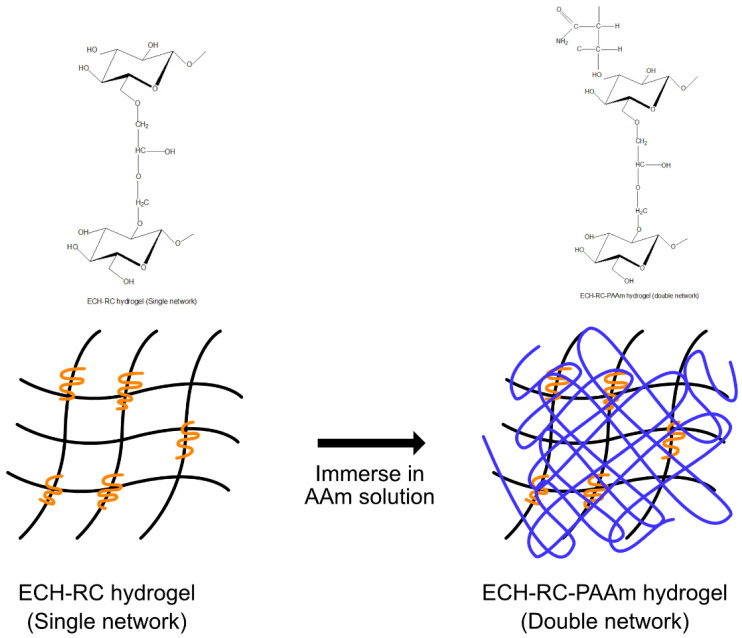
Single and double hydrogel network modified by PAAm.

The RC hydrogel is a sustainable initiative due to its biocompatibility and biodegradability has been employed in myriad applications such as controlled- and slow-release of materials, medium to retain water, and bio-sensors that are appropriate for agricultural purposes [58]. RC hydrogel can be produced via facile manners as it is compatible with a wide range of polar and non-polar solvents as well as compatible with other materials to increase the fabricated products [59]. The process of producing RC hydrogel includes few steps; (i) cellulose dissolution, (ii) regeneration of cellulose solvent in coagulant, (iii) crosslinking, and (iv) drying (for aerogel, cryogel, and xerogel productions). Besides, it is flexible to be physically or chemically crosslinked, as reported in numerous studies. Regardless of the excellent properties an RC can provide, it is unstable to form a product alone due to inter- and intramolecular hydrogen bonding, while its excellent porosity can cause shrinkage during regeneration, fragile, and higher elongation at break [60]. Thus, other materials will be incorporated into the RC system in order to increase the lack of characteristics. Furthermore, as mentioned previously, the crosslinking method also plays a crucial role in determining the end result of the fabricated RC hydrogel. It is flexible with either chemical or physical crosslinking (or both) approach, the properties of RC hydrogel could be adjusted accordingly, such as poor mechanical strength and toughness that limit its application [61]. For instance, a dual crosslinking network (chemically crosslinked by cinnamoyl-gelatin and further UV-photocrosslinked) that is applied to RC hydrogel with gelatin composite (dissolve together in 1-ethyl-3-methylimidazolium diethyl phosphate IL (EMIM)(DEP)) has better properties rather than chemically-crosslink the hydrogel only [59]. This dual crosslinking RC hydrogel has a higher compressive modulus and excellent water retention compared to hydrogel without photocrosslinking. In another study, single network (SN) RC hydrogel (ECH-crosslinked) and double network (DN) RC hydrogel (epichlorohydrin (ECH) and polyacrylamide (PAAm)-crosslinked) is compared [61] (Figure 4). According to this, the thermal stability, transparency, compression test, and elastic modulus increases on DN RC hydrogel. This study highlights the flexibility of RC in crosslinking as it has large –OH groups and the intermolecular hydrogen bonding forms chemical bonds with ECH that create spaces between them. The spaces perpendicularly increase with a higher ratio of ECH to cellulose, and this is an excellent property to develop a porous network of large water absorption; however, the mechanical strength and stiffness will lowered. Besides, the addition of PAAm into the system has created many crosslinking points and increase the mechanical and compression strength of RC hydrogel that in the same time reflects the flexibility of RC to form a homogenously blend with other materials. Apart from that, ionogel is fabricated from dissolution cellulose in IL and forming gel due to strong hydrogen bonds between regenerated cellulose and IL during regeneration process [62]. Ionogel is appeared to be yellowish due to dissolved cellulose and eventually develop into colourless gel during swelling [63]. The properties of ionogels include electroactive, better transparency and higher compressive strength compared to hydrogel.

The porosity of RC hydrogel could be changed amid the liquid removal or drying, which then produce aerogel, cryogel, and xerogel that depend on the desired interests. The supercritical- and freeze-drying of RC aerogel can retain the solid nano-porous structure; however, it gives fibrous aggregation uneven pore and lamellar aggregation as well as huge structure collapse [64,65]. The freeze-drying technique also leaves RC aerogel, cryogel, or xerogel to have points of the dense region at the RC rich regions because the ice crystals that form at these particular regions (during freezing) assemble into aligned columns and later will be squeezed by drying [59]. Then, they construct the RC fibrils to form an oriented cellulose scaffold. Other materials are incorporated with the RC aerogel production, such as silica, to prevent its structure collapse because the RC-rich regions will shrink after dried [66]. On the other hand, Ciocalu et al. (2016) had conducted a study to compare the type of crosslinking effect on hydrogel and cryogel from RC [67]. Chemical crosslinking by using ECH has shown less shrinkage on the RC products because the epoxy ring of ECH is actively reacting with the extensive –OH groups in the RC and is more homogenous; thus, it provides better swelling properties for both RC cryogel and hydrogel. This study also highlights the ‘hornification’ phenomenon during the transition of RC hydrogel to RC cryogel that resulted in less swelling in RC cryogel despite having better humidity adsorption. Limited studies of RC xerogel have been reported due to extreme volume shrinkage upon drying; however, research application is more inclined towards cellulose-xerogel that is derived from cellulose nanofiber (CNF) [68]. Buchtova and Budtova (2016) had shown that RC xerogel that is produced from low-vacuum evaporative drying on pore walls has very high volume shrinkage due to strong capillary pressure alongside densification due to large –OH in the RC.

**Table 3:** Extended products from hydrogel fabrication.

Type of Product	Method of Production	Properties	Appearance
Aerogel	First developed by Kristler in the 1930s performed by a supercritical drying to remove liquid from hydrogel [69]	Low density, large surface area, excellent mechanical properties, and high porosity. Sometimes, brittle aerogel is produced due to the drying technique applied. Commonly as an adsorption material.	White, opaque, lightweight
Cryogel	Hydrogel is frozen and lyophilizes at very low temperature to remove liquid	Lightweight and low density. Compact structure and large porosity depend on freezing temperature and ice crystals formation during freezing.	White, opaque, lightweight
Xerogel	Low-evaporative or vacuum drying of hydrogel and subsequently forming a thin porous film with extreme shrinkage [70].	Mimicking aerogel but improved in brittleness characteristic that is commonly generated from aerogel [68]. Dense, compact, and very low porosity.	Yellowish, shrunk, translucent

### 2.2. Fibers

Fibers are defined as thin thread structures made from natural or man-made materials. Fibers are divided into three primary groups; (1) natural fibers, (2) natural/man-made, and (3) man-made fibers. The natural fibers are cheap, low density, biodegradability, abundant, and have renewability properties [71,72]. They are classified into three main types such as plant fibers, which are known as cellulosic or lignocellulosic fibers, animal fibers, and mineral fibers. There are six groups under plant fibers such as leaf, bast or stem fibers, fruit or seed fibers, wood, stalk, and grass or reeds [73]. RC is categorized under the natural/man-made group. Three types of RC fibers such as modal, viscose, and lyocell. Meanwhile, man-made fibers are synthetic fibers like carbon, glass, and aramid [74].

Synthetic fibers have been commonly used in the various industries especially in the textile industry, but it offers a serious threat to the environment such as water pollution, and the source of synthetic fibers is made from petroleum. Water pollution occurs due to the procedure of making synthetic fiber like washing, bleaching, and dyeing that release various unwanted chemicals to the environment [75]. Hence, it is not preferable to fiber compared to the naturally made fiber, RC fiber which is biodegradable and safe to the environment. In addition, the source of raw material for making RC fiber is inexpensive, abundant, and has become the best alternative for substituting conventional synthetic fiber. Nevertheless, RC fiber still has drawbacks, such as the mechanical properties are poor when it is used alone. For that reason, there are few studies have been done on the enhancement of properties of RC fiber to use them in various applications [76]. Meanwhile, RC fiber also can be incorporated as reinforcement in the green composites [77,78]. The advantages of RC fiber compared to synthetic fiber as reinforcement are delicate and low density [79].

**Figure 5:**
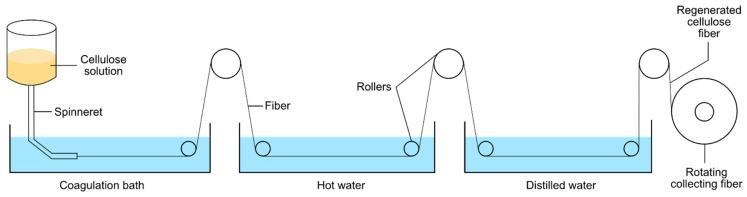
Schematic diagram of basic RC fiber processing.

There are few methods for producing high-quality RC fibers by dissolution and regeneration process [80]. Usually, the fibers will be prepared via the spinning method [34,81,82]. The RC fiber was obtained by coagulation process in which equipment consists of a polymer cylinder reservoir, spinneret, coagulation bath, and rotating fiber collector, as illustrated in Figure 5. The wet spinning technique is one of the common methods used in the formation of RC fiber. The fibers are made by extruding cellulose solution through a spinneret into the acid bath for coagulation. Next, the fibers are cleansed by utilizing hot water first and distilled water to eliminate the salts obtained during the coagulation process. The produced RC fiber is then dried at the ambient temperature [32,83]. Briefly, RC fibers can be employed in the agriculture industry due to the chemicals used for the production are low cost, non-toxicity, and environmentally friendly [83,84].

Different solvents systems could affect the properties of RC fibers. There are diverse inventions of solvent systems in the past research. Hence, few studies of RC fiber dissolve in different types of solvent and coagulation baths are tabulated in Table 4. Each solvent system could affect the final products of RC fiber and have its advantages. For instance, ionic liquids have been proven safe to be used, simple and these solvents enhance the tensile strength of RC fiber [85]. The rheological behavior of most cellulose solutions is the shear-thinning property and non-Newtonian flow [86]. This is due to the increasing amount of cellulose affect the molecular chains of cellulose to be increased [87]. The parameters of draw ratio, spinning dopes, and fiber spinning can be tailored to get the best structural and mechanical properties of RC fiber. According to Ma and the co-workers, the morphology of RC fiber will be irregular and severe grooves of the surface when the draw ratio is low. Meanwhile, the RC fiber form at a high draw ratio will obtain a smooth surface with a round structure [88]. Yang et al. have been discussed that the higher tensile strength has been contributed by the high degree of crystallinity and low orientation of microvoids [83]. The longevity of RC fibers is important to use for a long time. Therefore, the biodegradation of RC fibers should be monitored, and it is suggested to utilize the biodegradable polymer and combine it with cellulose to form composite RC fiber.

### 2.3. Membrane and Thin Film

RC membrane is also obtained via the dissolution-regeneration process and subsequent casting in coagulant and resulted in a microporous structure with hundreds of nanometers in diameter [91,92]. The fabrication of RC membrane and RC thin film is performed by the casting of the dissolved cellulose solution on a glass plate to form a uniform thickness of the membrane, whereas immersion of the glass plate in coagulant creates the regeneration of cellulose [93,94,95]. The research interest on RC membrane and a thin film is vastly due to its excellent mechanical properties, and high porosity has enabled it to be widely employed in the agricultural sector [96,97]. The ability to tailor the characteristics of membrane aids in a material with better resistance to severe cleaning mechanisms such as oil contamination treatments in wastewater treatment in which the treated water can be applied for irrigation purposes [98]. The inadequacy of clean water is a widely debated issue in the status quo due to the growing worldwide population, especially in continents such as Asia and Africa [99]. The agriculture sector will remain the largest user of clean water since it utilizes about 70% of global water for irrigation purposes [100]. A similar concept can be utilized during the application of cellulose membrane to separate oil from water, and this is one of the reliable measures to produce clean water, which can then be further utilized in agriculture.

**Figure 6:**
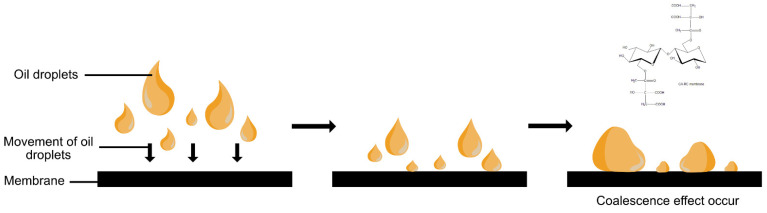
Formation of the hydrated sheath on RC membrane with CA crosslinking.

Traditionally, contaminated wastewater is cleaned up through some techniques such as flotation, adsorption, or coagulation that is gravity-driven for oil/water separation [101]. However, these chemical and energy-intensive techniques are expensive and have invited secondary pollution due to toxic and corrosive materials used [102]. Besides, the practicality of these techniques only lies in oil droplets’ size < 150 µm or between 20 to 150 µm for unstable oil droplets [101]. Concerning environmental pollution, the highly porous, hydrophilic, and oleophobic in water atmosphere cellulose is utilized as the oil/water separation-RC membrane [103]. The wettability under oil/water is essential in oil/water separation membranes due to its application in a wet state. The RC membranes also demonstrated high oleophilic properties when they formed a continuous layer of oil on the membrane’s surface and behaving as a water repellent, which is essential to separate the oil/water emulsions. Similarly, Fan et al. (2018) fabricated an all RC composite membrane with citric acid (CA) as the cross-linker and subjected it through an emulsion of oil/water [104]. They found out that the carboxyl groups from CA and hydroxyl groups from RC formed a hydrated sheath via capturing water molecules that gave a cushion-like behavior to the membrane that prevents direct contact with oil (Figure 6). Due to the low oil adhesion of the membrane, the oil droplets were able to roll on the membrane’s surface and became bigger due to the coalescence effect. The membrane achieved the removal of oil from water through the mechanism of filtration. The mechanism in both membranes fabricated by Fan et al. (2018) and Yagoub et al. (2019) was different, whereby the former utilized the concept of wettability and the latter utilized the concept of flotation and filtration [104,105]. The utilization of cellulose membrane in producing clean water can be further utilized in areas that require clean water for irrigation purposes.

## 3. The Potential Applications of Regenerated Cellulose Products for Agriculture

### 3.1. Excellent Water Conserver for Plantation Media

Several soilless agricultural systems have been introduced to this day, such as aeroponic, hydroponic, nutrient film technique (NFT), floating system, wicking system, drip technique, flow system, and aquaponics [106]. Soilless agricultural or soilless farming brings the ability to reduce environmental damage and a reasonable opportunity for the world to achieve sustainable agriculture [107,108,109]. In respect to this, few aspects that construct the soilless system cultivation make it promising through nutrients recycling to reduce the fertilizers’ run-off and vertical or stacked plantation to reduce the plantation’s space besides being soil-free [110]. Recently, the soilless plantation system that uses RC products has drawn much attention due to its promising applications for agriculture purposes. With many interventions that have been made in the past decades to rescue future generations’ needs, especially in the perspective of agriculture, growing soilless culture are being developed and implemented. Until these days, improvement is continuously pursued. The revolution of RC products (e.g., hydrogel-based products) that possess amphipathic properties, excellent biocompatibility that can be incorporated with diverse materials as well as biodegradable at a certain period have always encouraged researchers to ameliorate the intended products. RC hydrogel has shown much significance in the agriculture field, such as (1) aiding in moisture preservation that is exceptional for arid places and countries that undergo winter season as well as reducing water consumption in the conventional irrigation system, (2) a medium that can be loaded with nutrients and accommodate with controlled-release behavior mechanism, (3) maintaining and increasing soil health by reducing soil erosion and binding the loose soil to increase the soil grip for better root latching [111]. Few examples are listed in Table 5.

Increasing demand for food commodities is causing the expansion of agriculture and inciting greater water consumption due to conventional agriculture that uses irrigation systems. This circumstance forces high water usage on a global scale, and arid countries will overburden their water availability and transpire water scarcity. Water scarcity is defined as the demand for freshwater that exceeds its availability [117]. The potential of the RC hydrogel to be used as the plantation medium comes to light subjugated on its properties of porosity and retain water as well as swelling ability that gives rise to the better oxygenation for plants’ root due to porosity surge. Due to its excellent water retention ability, the hydrogel is also utilized to absorb and reserve the rainwater or irrigation water, which eventually helps in conserving water [118]. Cellulose is comprised of crystalline (highly ordered regions) and amorphous (less ordered regions) domains intertwined with the matrix of lignin and hemicelluloses subjected to its sources in dictating their compositions. The accessibility of reagents towards cellulose is limited because of its biphasic properties and favorably attacked the amorphous regions. However, various cellulose solutions are being explored [119]. The regeneration process of cellulose I produce cellulose II or amorphous cellulose has provided a better setting for reagents’ penetration to the cellulosic fibers whereby, for agriculture purposes, refers to water [120,121]. Water molecules can penetrate the cellulosic fibers attributable to the presence of the hydroxyl groups along the chains of the amorphous cellulose, forming hydrogen bonds [122]. The anisotropic van der Waals forces that exist in the cellulose chains also enhance the forces for strong adsorption energy to hold the water molecules as well as electrostatic interactions.

The interest in using hydrogel in future agriculture is confined to the soilless plantation; however, as the water conserver integrates it with the soil. Intending to water conservation, hydrogels with superabsorbent properties or known as SAHs, are being pursued [123]. Ample research on the modification to increase the strength of SAHs has been pursued because high hydrophilic chains contribute to high water absorption capacity and porosity as well as reduce soil erosion; however, this modification has loosened the structure of the hydrogel. Besides, the weak-gel construction is relatively unsuitable to support the root structure. As a water conserver, a hydrogel that is fabricated from rice straw as the source of cellulose has shown great water absorbance capacity. Hence, it helps reducing water irrigation values, irrigation frequency and strengthens soil aggregation [118]. Demitri, Scalera, Madaghiele, Sannino, and Maffezzoli (2013) showed the mixture of soil and hydrogel with a higher concentration of cellulose-chemically-crosslinked-hydrogel powder exhibits a triplicate water retention capacity tested on the cherry tomato cultivation [124]. Anionic carboxylated-cellulose hydrogel shows higher carboxyl groups in the system with suitable carboxylate content has shown excellent homogenous macroporous structure that enables the hydrogel to retain a good water capacity, subsequently promoting all seedlings growth [125]. Cellulose is also blended with other natural polysaccharides forming a composite that provides strength to the hydrogel while preserving the microporosity and water absorption [113]. The strength provided by the cellulose is suitable for the hydrogel as the plantation medium in conserving water for future soilless farming. Biodegradable synthetic polymers are also employed to be blended, such as grafted poly(acrylic) acid with cellulose to form SAHs that show high swelling capacity due to large pore volume observed due to electrostatic repulsion [115].

### 3.2. Long-Shelf Life Plantation Media

When cellulose is regenerated by the dissolution-regeneration process, a lower degree of crystallinity is obtained, reflecting the high amorphous regions as well as a significant increase in porosity [39]. Crystalline cellulose holds strong interchain hydrogen bonding between adjacent cellulose chains alongside weaker hydrophobic interaction that enacts insolubility and less accessibility [126]. During the dissolution process, the crystalline region of cellulose is disrupted leads to an opportunity for the microbes to cellulose degradation due to its high accessibility property for specific bacterial-enzyme in degrading the polysaccharides chains. Cellulase is known as the specific enzyme functioning to break down the cellulose that converts the cellobiose to glucose molecules [127]. There are two types of cellulases known as endocellulase, and exocellulase or cellobiohydrolases, where the former cleaves the internal bond, and the latter degrades the reducing and non-reducing ends of cellulose chains [128]. For instance, RC hydrogel used as the crop plantation medium must have a long degradation time to sustain the plantation for up to four months. Cellulose itself lacks antimicrobial property; ergo, upholding a long degradation time is becoming a challenge. Researchers have invented antimicrobial RC products by impregnating or mixing positively-charged antibacterial agents such as quarternary ammonium compounds into the fabricated products; however, leaching of the antibacterial agents is obtained [129]. Alongside some common non-biodegradable and biodegradable, antibacterial agents have been employed to fabricate antibacterial RC products such as N-halamines, metal oxides, halogenated compounds for the former, and chitosan, alginate, curcumin, allicin for the latter. Cellulose is very flexible due to the abundant hydroxyl groups it possesses for excellent modification, such as antibacterial RC products.

Suitable antibacterial agents must be able to withstand certain temperatures and conditions to be incorporated into RC products, such as during cellulose dissolution (specific temperature applied) or regeneration [130]. As an example, epsilon-poly-L-lysine (EPL), a broad-spectrum antibacterial agents, is used alongside the fabrication of RC beads [131]. Although EPL is stable in both acidic and alkali conditions, it has a leaching problem that can cause contamination; thus, an excellent material such as RC beads is a good candidate to covalently bond with EPL. Alongside, incorporation of EPL into RC beads matrix is shown to have good microorganisms inhibition compared to RC beads alone as well as slower biodegradation rate compared to RC beads. Besides, the high porosity of RC beads has shown the enhancement of antibacterial activity of EPL due to large surface area for grafting oxidation of EPL [132]. Furthermore, the incorporation of chitosan into RC products has shown positive effects on plant growth, antibacterial, antioxidant, and antifungal activities [133]. Chitosan possesses a similar structure to cellulose, where the difference is distinctly observed in the amine group at C_2_. A similar structure drives chitosan and cellulose to have a homogenous blend in forming RC products by using the same cellulose solution system as reported in few studies [134,135]. The synergistic between chitosan and endophytic bacteria (soil bacteria) such as *Bacillus thuriengensis* has shown a reinforcement in fungicidal and insecticidal activity due to the presence of chitin-binding protein inside the particular strain [133].

### 3.3. Nutrient Reservoir

Slow-release fertilizer (SRF) and controlled-release fertilizer (CRF) allow nutrient availability for a longer time by a gradual release of fertilizer compared to the standard fertilizer used in conventional agricultural systems. These improved fertilizing methods reduce nutrient run-off and terminate the need for split application, which incurs cost [136]. The term SRF and CRF are being used interchangeably; however, they portray some differences in fertilizer release mechanisms. Ramli (2019) reported that there are three types of SRF, (1) matrix-type formulations, (2) coating-type, and (3) chemical products [137]. SRF can be formed into granules or tablets, which can be covered by using hydrophobic polymers or matrices and only concerned with the rate of nutrient release at a slower rate while there is no control on the release rate, duration, and patterns driven by environmental conditions such as soil and climate [138]. On the contrary, CRF allows the release of fertilizer based on pattern, duration, and rate of release with several types of formulation listed as (1) organic nitrogen-low-solubility compound, (2) inorganic low-solubility compound, and (3) hydrophobic coating polymer [139]. The similarity of the formulations for both SRF and CRF is the usage of coating polymer to slow/control the release of the fertilizer. Besides, this particular formulation is best represented for agriculture purposes. Coating the fertilizer using polymer will give the immobilization effect, thus, reduce run-off and leaching [140]. The protective coating regulates the water penetration and causes dissolution and nutrient release rate to coincide. Cellulose is best employed as the coating polymer for the SRF, and CRF is subjected to its biodegradability, inexpensive, non-toxic as well as possesses hydrophobicity and hydrophilicity [141,142]. The high porosity of RC products has made them suitable for releasing mechanism ability or pictured as transport channel [129].

SRF-hydrogel (SRFH) could be derived from natural or synthetic via matrix or coating-types and found to hold water retention and SRF ability. By considering this, RC hydrogel has become the center of attention for researchers in developing SRF for the agriculture industry. Ramli and co-workers used coco peat fiber as the cellulose source to produce RC-SAH via *in-situ* solution polymerization technique grafted-poly(acrylic acid)/Nitrogen-Phosphorus-Potassium (NPK) SFRH [143]. The coco peat fiber-SRFH showed a homogenous network, thick and smaller pore sizes that limit the water penetration to reduce the fertilizer release up to five weeks and concur an excellent mechanical property. Zhang and co-researchers prepared hydrogel based on sawdust cellulose-SRF by graft copolymerization and showed a greater water holding capacity due to the introduction of hydroxyl groups and mutually slowed down the fertilizer release in the early and late-stage [144]. The primary methods to produce CRF are using water-insoluble, semipermeable, or impermeable-with-pore polymer coating [139]. The release mechanism is affected by the hydrogel network’s gel strength, where lower gel strength leads to an increase in the release rate [144]. There are a few factors that determine excellent SRF/CRF-RC-coated, particle size, gel strength, and porosity. According to Olad and co-workers, the release rate of fertilizer in the SRF was rapidly at the beginning, but as time is increased, the rate of release tended to be low [145]. Wheat straw cellulose hydrogel showed high porosity and small particle to hold water that allows slow release of fertilizer [146]. The high porosity of SRF/CRF-RC-coated reduces the release rate of fertilizer as Aini et al. (2019) showed large pore of rice straw cellulose/urea beads had caused an increase in the urea diffusion rate [147]. Pores promote water diffusion into the network, which results in more water absorption and increases the swelling rate of hydrogel [143]. Therefore, the hydrogels formed should have small porosity and high gel strength to ensure the release rate for fertilizer decreases.

**Figure 7:**
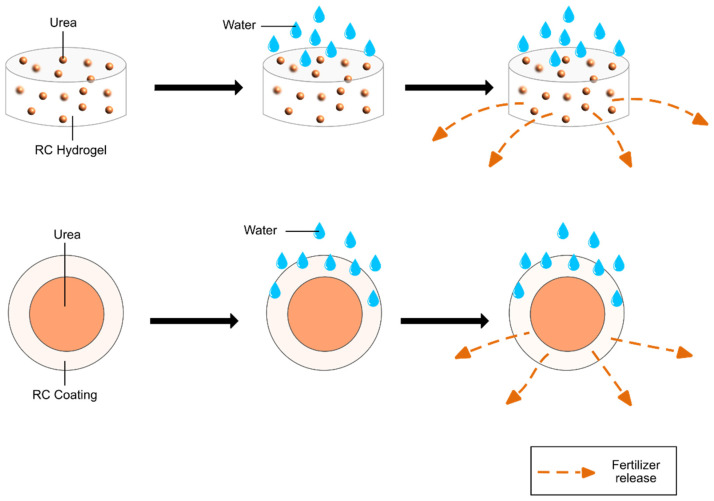
Illustration of RC hold/coat urea fertilizer.

Figure 7 displays an illustration of RC hydrogel hold or coat the urea fertilizer. Li and the co-workers discussed that RC hydrogel is crosslinked hydrophilic polymer networks, which allowed the penetration of water to occur in the capillary action and hydrophilic group dissociated from polymer chain resulted. The phenomenon is called water absorption of the hydrogel. Fertilizer enclosed in the hydrogel will be diffuse and begin to disperse as the difference of concentration gradient between outside and inside hydrogel. Hence, the exchange of water in hydrogel and fertilizer takes place [148]. There are steric effects, and adsorption of fertilizer will be blocked the dissemination of fertilizer. Hence, the nutrients released will be varied and slowed down [146].

The kinetic release from various types of RC hydrogel in the soil has been collected and tabulated in Table 6. Few mathematical models can be used to determine the kinetic release study and the table contain information of correlation coefficient (*R*^2^), which determines the finest fertilizer release study. The release mechanism applies to Fick diffusion when *n* less than or equal to 0.45 while between 0.45 and 0.89 indicates anomalous transport and when greater than or equal to 0.89, it shows as release mechanism of Case II [149,150]. As when the curve is high goodness of fit (*R*^2^ > 0.96), the result reveals the model is valid to be employed because the rate of fertilizer release relying on concentration [151].

There are some drawbacks and limitations to using RC as a nutrient reservoir. RC products only cannot retain water properly and do not effective as nutrient reservoirs. Synthetic polymers like polyvinyl alcohol (PVA) have exceptional characteristics such as excellent hydrophilicity, biodegradable, and non-toxicity [154]. Despite that, PVA cannot form the outstanding hydrogel in the aspect of strength and low swelling ratio for use as fertilizer release [155]. Hence, the incorporation of other polymers with cellulose as RC hydrogel for nutrients’ reservoir is favorable as to build up the advanced properties of fertilizer release mechanism.

### 3.4. Protective Materials

Mulching can be defined as the spreading of organic or inorganic materials on the soil surface that form a physical barrier that protects crops from soil contamination and maintains the soil’s structure [156]. Mulching materials can be divided into organic materials that are derived from animal and plant-based such as coconut husk, rice straw, and cow dung, whereas inorganic materials are synthetic materials derived from petroleum-based products such as polyethylene plastic films [157,158,159]. Plastic mulching has been reported in several preliminary studies to improve the water irrigation performance in agricultural lands that faces drought and is extensively used in agriculture due to its outstanding abilities, such as high hygroscopicity, which improves the soil moisture, temperature as well as enhancing the yield of crops [160,161,162,163,164]. Chopra and Koul (2020) discussed the advantages and disadvantages of using mulches in their work. Although organic mulches are biodegradable and environmentally friendly, they decompose easily and require frequent replenishment in a short period [165].

Apart from mulching films, fruit protection nets also gained a broad research interest in agriculture due to their ability to reduce the amount of radiation received by the crops and also their capability to withstand abiotic and biotic factors that affect the quality and productivity of the crop [166]. The nets diffuse the solar radiation; hence, the crops are exposed to uniform light from all sides and further improve the production and the ripening process of fruits. Furthermore, agricultural nets can moderate the microclimate beneath the nets, which improves the quality of the crop [167]. The application of plastic film has been implemented since the middle of the twentieth century, and fruit nets were mainly utilized as a protection for fruits, to prevent the consumption of pesticide and herbicide, and to protect the crops from wind, rain, and hail [168]. The types of nets can be differentiated through their structural features such as the material of the thread, shape, and dimension of fibers and meshing; and physical properties such as the color of the net, durability, porosity; and mechanical characteristics such as tensile stress, strength, and elongation [169].

The quest to find a suitable degradable material for mulching films has been widely discussed in the research field [170,171,172]. RC has been prominently studied for its unique properties, such as biodegradability and remarkable mechanical and thermal properties [173,174]. Certain essential properties have to be taken into consideration for the application of RC as a mulching film, such as its mechanical properties and tensile strength to avoid fracture in its structure [175,176]. Apart from that, the membrane’s thermal stability is also important to ensure the prolonged thermal degradation caused by molecular deterioration. Although RC is a biodegradable material, the longevity of this material has to be maximized to reduce frequent replenishments of the mulching films to avoid additional costs.

Crop productivity in the agricultural sector that utilizes plastic mulching is highly dependent on the physical appearance of the mulching material. The effects of different colors of plastic mulches on the development and yield of tomatoes were compared in a study by Mendonça et al. (2021). They reported that dark-colored plastic mulches had an increase in soil temperature [177]. They attributed this to the ability of the mulch to absorb, and more shortwave radiation transmission and similar responses were recorded by Sarkar et al. (2019) [178]. Cellulose-based black mulch film (BMF) was fabricated, and its effect on the soil temperature and yield was studied. It was reported that the BMF depicted the least variance in the soil temperature, which was attributed to the smaller light transmittance and solar radiation [176,179]. They further elaborated that a suitable temperature is essential to enhance the growth of plants. The soil temperature is one of the most prominent factors in determining crops’ productivity and quality. The usage of colored degradable mulching film aids in creating a distributed soil temperature that promotes the growth of plants. Sintim et al. (2020) co-related the mulch degradation with thermal time in which they reported that the degradability of cellulose occurs only below 55 °C [180]. The degradation rate for cellulose was reportedly low during higher temperatures. This characteristic of cellulose can be further applied for plastic mulching in areas with higher temperatures to prolong the degradability rate. Owing to the properties above, the ecosystem of the soil can be preserved by reducing synthetic plastic mulching material, which causes negative effects to the soil, such as reducing the soil porosity and inducing plastic waste into the food chains [181,182,183].

### 3.5. Biosensors

Chemical- and bio-sensors have been employed in wide applications that convert the signal into interpretable information for products’ quality improvement, and environmental management is an electronic recognition system [184]. A Recognition system is often employed in many fields consisting of the agriculture sector, and immense improvement is being committedly performed for future smart farming. By considering this, sensors are continuously developed to monitor agriculture performance by analyzing few parameters such as soil humidity, heavy metal ions (HMIs) contamination, soil health and diseases, and fertilizer leaching. Cellulose is converted into RC products such as films and fibers because of its biodegradability, non-toxic as well as possessing great hydrophilicity properties. Cellulose-based pH indicator for ammonia detection is fabricated by mixing cellulose with activated dyes in solution and wash with distilled water as an alternative to the chemical sensors made from metal oxide semiconductor [185]. RC xerogel is developed as an ammonia detection sensor, has shown to exhibit low detection time and high sensitivity that makes cellulose-based sensor is a promising detection system due to the large surface-area-to-volume ratio and high micro-porosity properties subjected to the dense cellulose fiber network [70]. Chemical sensors that are developed from certain immobilization components commonly leach amid functioning into the detection environment, and this practice is unhealthy, especially in the agriculture sector, thus, by using RC products to covalently immobilize dyes as well as sustainable and biodegradable [185]. The ability of cellulose to immobilize dyes also exhibits a uniform dyes’ distribution on the RC film for high detection of heavy metal ions that are present in the irrigation water source [174]. Other than being an immobilization particle for the dyes employed in chemical sensors, cellulose is often being reinforced by other biodegradable synthetic polymers such as polyvinyl alcohol (PVA) to form flexible sensor film because of massive amorphous region of cellulose have aided in enhancing mechanical strength and optical transparency [186]. The humidity level of soil or moisture of the soil is a pivotal parameter to be monitored to yield quality crops. Volumetric water content and root zone leeching checking are usually probed to govern the optimal irrigation frequency influenced by the temperature, soil salinity, and different soil types [187]. The abundant hydroxyl groups in the RC structure make it a natural polymer with a high affinity towards water alongside its transparency and high porosity characteristic that shows that RC products are an excellent candidate for a humidity sensor [188].

## 4. Conclusions

Cellulose is one the most bountiful biomaterial found on Earth that is mostly extracted from the cell wall of plants, and tunicates are widely used in a variety of applications due to their unique characteristics such as good biodegradability that makes it environmentally friendly and green, great mechanical, good thermal strength and non-toxicity. Nonetheless, the application of cellulose is bounded due to its insolubility in water, and it is often counter-measured with the surface modification of the large surface area of cellulose. The dissolution-regeneration process has given cellulose a huge advantage for extensive modification subjected to the changes in the cellulose degree of crystallinity. The more amorphous or less ordered region in RC allows a variety of surface modifications to be carried out as compared to native cellulose. The abundance of OH groups in cellulose enables it to be tailored according to the applications such as in the medical field, paper industry, packaging, and food industry. However, the application of cellulose in agriculture is yet to be explored extensively.

This review has attempted to give a clear insight into different RC products and their utilization to improve the current conventional agricultural system. The application, as well as research and development of RC in agriculture, are limited at present; hence more resources to explore RC can be carried out to create a more sustainable world that encourages smart farming. The RC products discussed in this review have their own limitations and advantages in the perspective of mechanical strength, cellulose shrinkage after regeneration, and fast biodegradability. A variety of materials and techniques to construct the RC products for agriculture purposes are discussed, focusing on the homogeneity blending of RC with other materials. The blending shows a great potential to increase the end properties of RC products. The development of RC products has a great influence from the following element, (1) dissolution and regeneration solvent type, (2) source of native cellulose (3) type of materials that are blended with RC. A wide range of biodegradable synthetic and natural materials are shown to have good compatibilities with RC as well as exerting excellent properties on the end results. For the future prospect, critical studies on the effect of blending the additional materials before dissolution, during dissolution, during regeneration, or after regeneration on end properties of RC products could be conducted to understand the RC homogeneity in-depth to tailor with the desired applications.

## Figures and Tables

**Table 1:** Cellulose solvents and their action mechanism.

Type of Solvent	Name of Solvent	Formation of Intermediates	Reaction Mechanism
Derivatizing	*N*,*N*-dimethylformamide (DMF)/dinitrogen tetraoxide (N_2_O_4_)	Etherification reaction occurs at O6 and O2 that forms cellulose I into cellulose nitrite arose from N_2_O_4_ as the intermediate preceding the dissolution as DMF acts as the organic solvent. Common catalysts such as sulphuric acid or phosphoric acid are involved in this reaction.	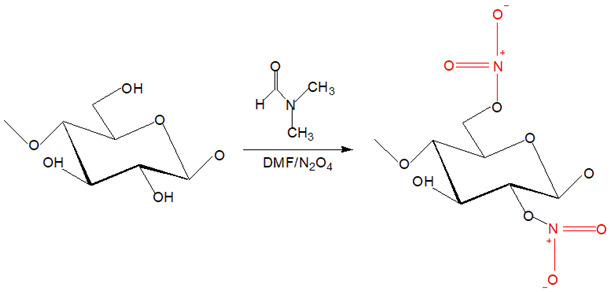
Derivatizing	Trifluoroacetic acid	Cellulose triacetate is formed through esterification reaction at O2 and O6 as derivatives before dissolution takes place.	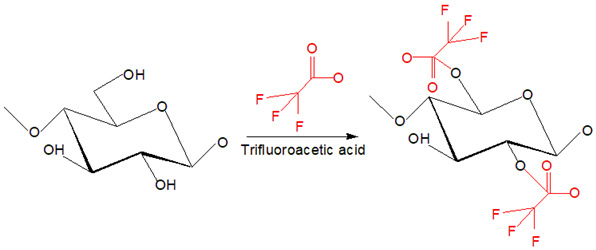
Non-derivatizing	*N*-methylmorpholine-*N*-oxide (NMMO)/water (aqueous system)	Widely used as the industrial cellulose solvent, the highly polar N-O bond is able to form one or two hydrogen bond(s) with two hydroxyl groups in cellulose. The molecule of H_2_O that is smaller than NMMO also aids in breaking the inter- and intramolecular hydrogen bonding [25].	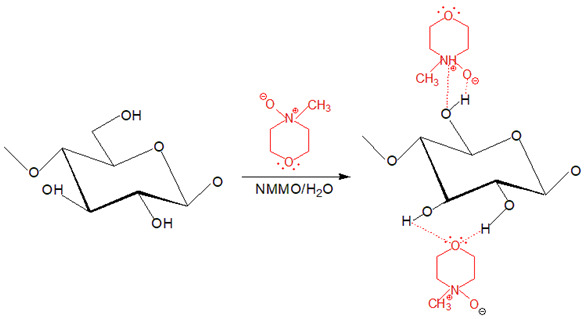
Non-derivatizing	Sodium hydroxide (NaOH)/urea/water (aqueous system)	Cellulose chain degradation is caused by alkaline hydrolysis. NaOH is interfering with inter- and intramolecular hydrogen bonding of O3H--O5′, O2H---O6′, and O3′---O6H during the process of mercerization of cellulose [37].	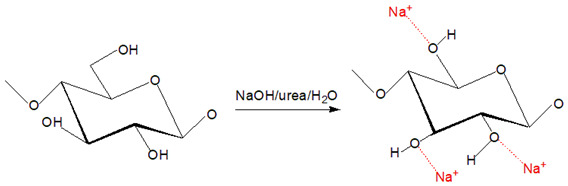
Non-derivatizing	1-butyl-3-methylimizadolium chloride [BMIM]Cl (non-aqueous system)	IL that is derived from cationic BMIM^+^ and anionic Cl^-^ caused the charges are distant due to bulky ‘shell’ around the BMIM^+^. Cl^-^ forms bonds with H^+^ in the hydroxyl group of cellulose to interfere with the supramolecular structure.	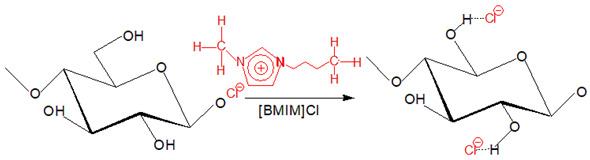

**Table 2:** The characterization of transformation of cellulose I to cellulose II via XRD, Fourier-transform infrared spectroscopy (FTIR), ^13^C NMR, and Raman spectroscopy.

Characterization	Method	Cellulose I	Cellulose II	Explanation
Crystalline structure	XRD	Crystalline interplanar (1–10), (110), (012), (200), (004) Miller indices and amorphous are represented by 12.3°, 16.5°, 20.4°, 22.6°, 34.4° and near 20.5° [42,43].	Crystalline interplanar of (1–10), (110), (020) and amorphous shown in XRD profile of cellulose II that are represented by ~12.3°, ~20.1°, ~22.0° and ~20.5°. Overlap (110) and (020) peaks might be attributable for more amorphous RC subjected to type and parameter of cellulose solvent [42,44].	The loss of peak and generation of the crystalline peaks are due to the rebuilding of hydrogen bonding [42].
	FTIR	1428 cm^−1^ peak is considered to be the crystalline region [45,46]	The 1428 cm^−1^ peak is lost [42].	
	^C^13 CP-MAS NMR	Singlet signal 100–110 ppm, 96 ppm, and 60–70 ppm correspond to C_1_, C_1_ (reducing end), and C_6_ [47]	Extra small shoulder peak at 107 ppm, peak of C_1_ reducing end at 96 ppm is not detected, and C_6_ peak is shifted [47]	C_6_ peak confirms the crystalline structure of cellulose, where, when it is not detected in cellulose II, it is shown that cellulose has a lower degree of polymerization compared to cellulose I.
Chemical structure	FTIR	O-H stretching, C-H_2_ stretching, and C-O-C pyranose ring vibrations at 3700–3300 cm^−1^, 2900 cm^−1^, and 1163 cm^−1^ [42,48]	O-H stretching, C-H_2_ stretching, and C-O-C pyranose ring vibrations at 3345–3393 cm^−1^ (shift to a higher number), 2921 cm^−1^ (shift to a higher number and 1157 cm^−1^ (shift to a lower number) [42].	Transformation of cellulose I to cellulose II has occurred. O-H peak is shifted to a lower number is due to more –OH are created and more water adsorption [48].
Decomposition	Thermogravimetric analysis (TGA)	T_onset_ that refers to the removal of water is higher while the decomposition of cellulose reflected by T_max_ is lower [42,49,50].	In contrast, T_onset_ that refers to the removal of water is lower while the decomposition of cellulose reflected by T_max_ is higher [42,49,50].	The lower T_onset_ is due to the lower crystallinity recorded in cellulose II. Cellulose II is more thermally stable than cellulose I is due to its more ordered antiparallel chain that is regenerated after dissolution.
	Differential scanning calorimetry (DSC)	Has higher T_g_ = 64 ℃ [51]	Lower T_g_ = 62 ℃ [51]	More –OH surface groups on cellulose I

**Table 4:** Properties of RC fiber in different types of solvents.

Source of Cellulose	Type of Solvent	Coagulation Bath	Properties of RC Fibers	References
Sugarcane Bagasse	*N*-methylmorpholine-*N*-oxide (NMMO) hydrate	Ethanol	Morphology: Higher fibrillation Elongation at break: 2% Tensile strength: 530 MPa	[89]
Dissolving pulp	1-butyl-3-methylimidazolium chloride (BMIMCl)	Water	Morphology: No obvious voids and intact surface with dense inner structure Elongation at break: 6.6% Tensile strength: 808 MPa	[90]
Wood pulp	1-butyl-3-methylimidazolium acetate ((Bmim)OAc)/dimethyl sulfoxide (DMSO)	Water	Morphology: Irregular, serrated cross-section and severe grooves of the surface Elongation at break: 5.4% Tensile strength: 22.5 cN/tex	[88]
Soft wood pulp	Tetrabutylammonium acetate (TBAA)/dimethyl sulfoxide (DMSO)	Acid	Morphology: Smooth surface as round and compact structure Elongation at break: 9.7% Tensile strength: 2.15 cN/dtex	[87]
Cotton linters	NaOH/thiourea/urea	Acid	Morphology: Smooth surface and circular cross-section with homogeneous and denser structure Elongation at break: 9.47% Tensile strenghth: 2.22 cN/dtex	[83]

**Table 5:** Example of the RC products in the agricultural potential uses.

Type	Material	Classification	Method	Crosslinker	Swelling Capacity	Porosity	Application	Reference
Hydrogel film composite	Cellulose/gelatin	Natural	Dissolution-regeneration process with IL and distilled water	Photocrosslinked by high UV at different time length	Higher with longer time length	-	High water retention ability	[59]
Superabsorbent composite hydrogel film	Microcrystalline cellulose/carboxymethyl cellulose/hydroxyethyl cellulose	Natural	Dissolution-regeneration process using cold phosphoric acid and regenerate by using water	Citric acid at 80 ℃	Approximately 4000%	-	Coating material for control release fertilizer	[112]
Hydrophilic Composite hydrogel	Cellulose/linseed gum	Natural	Dissolution-regeneration process with NaOH/urea and regenerated by distilled water	Epichlorohydrin at 60 ℃ 30 min	more than 330 g/g	28 µm	Water conserver to be mixed with soil	[113]
Hydrogel	Cellulose	Natural	Dissolution-regeneration process with NaOH/urea system	Citric acid at varied temperature	Increase with increasing cross-linking temperature, more than 20%	-	Cultivation culture media for plantation	[114]
Grafted SAH	Cellulose/polyacrylic acid	Natural-synthetic biodegradable	Dissolution-regeneration process by using activated cellulose which are then dissolved and washed in distilled water.	*N*,*N*-methyle-nebisacrylamide	More than 3000%	More open and loose structure	Soil optimization to increase efficiency in seed cultivation	[115]
Composite hydrogel	Cellulose/bentonite	Natural	Dissolution-regeneration process using NaOH/urea as a solvent and regenerate using distilled water	Epichlorohydrin	5.74 g/g	-	Promoting seed growth and adsorption in soil	[116]
Hydrogel	Wheat straw cellulose	Natural	Dissolution-regeneration process by dissolving in NaOH and regenerated using ethanol.	*N*,*N*-methyle-nebisacrylamide	3095.74% swelling capacity with intermediate cellulose concentration	-	Soil has high water retention and improving seed growth and germination	[54]

**Table 6:** Kinetic parameters of different types of RC hydrogels in soil.

RC Hydrogels	Source of Cellulose	Diffusional Exponent, *n*	Release Rate Constant, *k*	Correlation Coefficient, *R*^2^	Model	Nutrient Release (%) Per Time	References
Sawdust cellulose-grafting-poly(acrylic acid)-poly(acrylamide)-Urea	Sawdust	1.3214	0.0012	0.9781	Ritger-Peppas	2.4% per 10 min 8.6% per 30 min 82.4% per 480 min	[144]
Cellulose hydrogel-Urea	Cellulose microcrystal	1.4476	0.2296	0.9899	Ritger-Peppas	22.9% per 1 day 49.5% per 3 days 75.6% per 6 days 91.3% per 10 days 95.71% per 21 days	[152]
Salep-g-poly(acrylic acid)/montmorillonite clay and NPK	Salep	0.3371	0.3004	0.9887	Korsmeyer-Peppas	14.12% per a day 30.32% per a week 55.36% per a month	[145]
Sulfonated-carboxymethyl cellulose with acrylic acid in polyvinylpyrrolidone, silica nanoparticles and NPK	Carboxymethyl cellulose	0.3570	0.3423	0.9862	Korsmeyer-Peppas	14.6% per a day 27.6% per a week 54.6% per a month	[153]
